# Comment on Le Floch & Ropars (2017) 'Left–right asymmetry of the Maxwell spot centroids in adults without and with dyslexia’

**DOI:** 10.1098/rspb.2023.2060

**Published:** 2024-02-28

**Authors:** Florian Naudet, Mark Seidenberg, Dorothy V. M. Bishop

**Affiliations:** ^1^ Univ Rennes, CHU Rennes, Inserm, EHESP, Irset (Institut de recherche en santé, environnement et travail) - UMR_S 1085, Centre d’investigation clinique de Rennes (CIC1414), F-35000 Rennes, France; ^2^ Institut Universitaire de France (IUF), Paris, France; ^3^ University of Wisconsin-Madison, Madison 53706-1314, USA; ^4^ Department of Experimental Psychology, University of Oxford, Oxford OX1 3UD, UK

## 1. Introduction

In October 2017, *Proceedings of the Royal Society B* published an article by Le Floch & Ropars [1] claiming that dyslexia could be caused by a visual anomaly that led to confusion between images from the two eyes when reading letters. This article attracted considerable media attention, with an Altmetric score of 829, including coverage by 83 news outlets, and has subsequently been cited in promotional material for devices that are designed to ameliorate the problem. A number of international experts raised concerns about the study on the post-publication peer review site PubPeer [2], but this has not led to any moderation of the claims made for therapeutic implications of the study. Given that our view is that the study suffers from methodological, interpretive and ethical problems that should have precluded publication, we are grateful to the editors for providing this opportunity to document these issues.

## 2. The claim

The notion that dyslexia is a visual disorder in which there is confusion between letter identities has appeared in a range of versions over the last century, starting with Orton's [3] account of ‘strephosymbolia’. Such explanations declined in popularity as a growing body of research from 1960 onwards showed that dyslexia is characterized by a linguistic deficit affecting phonological processing; the dominant position is that visual symptoms in dyslexia are more likely to be a consequence rather than a cause of reading difficulties [4,5]. Even those that recognize ‘visual dyslexia’ are clear that this is one of many subtypes. As Friedmann & Coltheart [6] noted: ‘whereas visual impairments can impede reading, not all dyslexias result from visual impairments. Moreover, even the dyslexias that result from an orthographic-visual analyzer impairment do not result from a visual or attentional impairment. It is often the case that the impairment is selective to orthographic material and does not apply to reading numbers and symbol sequences'. This does not, of course, rule out the possibility that the consensus could be flawed, and/or that a hitherto unrecognized form of visual anomaly might cause or contribute to dyslexia in a subset of individuals. It does, however, set a high evidential bar for any new theory that proposes a purely visual basis for dyslexia.

Le Floch & Ropars [1] proposed a novel version of a visual theory of dyslexia, with a specific mechanism relating to symmetry of sensory regions of the fovea, claiming that two tests confirmed the putative deficit in 100% of a sample of dyslexic adults, and in 0% of a healthy control sample. Such perfect correspondence between diagnostic status and test results is unprecedented in our experience, and so would be a truly remarkable discovery if replicated (table 1).

**Table 1.** Distribution of dominance on two measures of asymmetry: foveascope (first letter) and afterimage test (2^nd^ letter). L = left, R = right, N = no dominance.

	LL	RR	LR	RL	NN
typical adults	11	19	0	0	0
dyslexics	0	0	1	2	27

The evidence came from two methods. First, observers were tested using a new device, the foveascope, which projects bright white light onto a screen through a blue-green exchange filter. When a green filter is applied, the observer sees a lighter green zone on a dark green background: this corresponds to a region in the centre of the fovea that lacks blue cones. By testing each eye separately as the apparatus changes from a blue to green filter, the size and shape of this area, known as the Maxwell spot centroid, can be identified, by having the observer draw the outline of what they see on the screen and comparing it with the projected image. A second dependent measure is ‘noise-stimulated negative afterimage dominance’, a measure of eye dominance. Both eyes fixate on a stimulus, such as a yellow ‘x’ on a bright screen, and are then closed. This creates an afterimage (i.e. an x remains visible in the absence of the stimulus). The dominant eye is defined as the one where the afterimage takes longer to fade. In typical adults, 63% were right-eye dominant and 36% left-eye dominant, and there was 100% agreement between dominance assessed this way and the blue cone-free area asymmetry. However, for 30 dyslexic adults, only three showed eye dominance on the afterimage test (two left and one right). The 27 who showed no eye dominance also showed no asymmetry on the foveascope test. Furthermore, the three who did show eye dominance also showed asymmetry on the foveascope, but in the opposite direction. This was explained post hoc by the fact that early in development they had strabismus, severe amblyopia or a facial malformation leading to ‘frustrated afterimage dominance’.

## 3. Methodological issues

### (a) Characterization of the sample

Le Floch & Ropars's study [1] compared 30 dyslexics with 30 healthy controls, both groups being drawn from the university student population. The only information provided is that dyslexics had been examined by ‘the medical staff of the university’ and all ‘encountered difficulties in reading, spelling, writing and recognizing left from right’, and were accordingly given 30% extra time in examinations.

A study on dyslexia would normally include descriptive data about the subjects in the two groups to establish the validity of comparisons between them, including age, handedness, language background, a measure of nonverbal IQ, measures of spoken language such as vocabulary and comprehension. Then there would be assessments using reading-related tasks on which dyslexics are typically impaired, such as reading words and nonwords aloud, judging which of two stimuli is the correct spelling of a word (e.g. RAIN RANE), speed and accuracy of naming letters, colours, objects. In French, which has more consistent spelling–sound correspondences than English, dyslexics would be expected to read words and nonwords aloud accurately but slowly, with a bigger impact of word length than in English [8]. Without specified criteria for diagnosing dyslexia, the study is impossible to replicate. In addition, the sample is a mixture of students with various difficulties. For instance, all included participants had difficulties in writing and recognizing right from left. Many dyslexics have normal writing and do not suffer from letter orientation problems. It is therefore impossible to confirm that these subjects are really dyslexic as defined by at least an objective and independent measure of reading speed and accuracy, and some comprehension test. The authors also need to state whether those diagnosing dyslexia regarded a mismatch between handedness and eyedness as a marker for dyslexia: this was not uncommon in the twentieth century in centres influenced by Orton [9]. If this applied here, then the sample would be biased.

### (b) Potential for bias in testing procedure

Le Floch & Ropars's text [1] states: ‘we tested a first cohort of 30 healthy students […] we performed the same tests with a second cohort of 30 students with similar visual status and optical capabilities as the first cohort, but suffering from dyslexia’. The natural interpretation of this account is that the two groups were tested sequentially. This would create a serious risk of experimenter bias and/or apparatus bias. Regarding apparatus bias, the foveascope is a not a standard piece of laboratory equipment and there is no information on how far its results are subject to systematic or random error; the best way to control for such bias is by avoiding any confound between time of testing and observer group. The usual way of dealing with experimenter bias is to ensure that the researchers gathering the data are unaware of which group the participants belong to [10]. This is particularly important when the dependent measure is subjective report, where responses are likely to be influenced by demand characteristics [11]. When the issue of sequential testing was raised by us with the editor of the journal, the authors replied to the editor that both cohorts were ‘tested randomly according to their respective availability’. This is confusing as ‘random’ does not mean ‘according to their respective availability’, and the statement also seems at odds with the account in the paper.

### (c) Lack of clarity about experimental procedure

Le Floch & Ropars's accounts of how measures are taken from the foveascope and the afterimage test are not easy to follow. Our understanding is that for the foveascope, the observer views filtered light projected onto a screen and draws on a tablet the outline of the differently coloured area that they see in the centre of this screen. The participant's drawing is simultaneously projected onto the same screen, to ensure that it overlaps with the differently coloured area (i.e. what is drawn should be a representation of the Maxwell's spot centroid, the blue cone-free region). This is done for left and right eyes separately. The statement that ‘each observer adjusts the modulation frequency between 0.1 to 1 Hz to his best convenience’ seems to refer to the shift in the blue-green filter, but suggests uncontrolled variation in how stimuli were presented. For each eye, the ellipticity is assessed by fitting an ellipse around the drawing and measuring its width relative to length—for a circular drawing, ellipticity is one, whereas if the length of the drawing was twice the width, it would be 0.5. Asymmetry is measured by subtracting the ellipticity measure for left from the right. The two representative examples shown by Le Floch and Ropars have a circular centroid on one side and an elliptical one on the other.

It is evident that the dominant centroid according to this method is not necessarily the one with the larger area—the reason for using ellipticity as the dependent variable is not stated.

Also missing from the experimental methods is any information about instructions to the participants, whether each person had a single trial with each eye or whether multiple trials were given.

The afterimage test is illustrated with a video in supplementary materials [12]. Again, no information is provided regarding instructions to participants, even though the dependent measure is subjective report of whether or not an afterimage is present. It is not clear whether repeated measures were taken. There is a mixture of methods described in the text and in the video, including binocular testing, monocular testing, and testing with different stimuli, but it is not clear which of these provided the basis for the data in their fig. 2.

## 4. Failure to consider prior literature

As noted above, there is a very long history of studies of eye dominance in dyslexia, but most of this is ignored by the authors. It could be argued that their methods are novel and so the prior literature is irrelevant, but where they do use traditional measures, the striking group differences are out of keeping with prior literature. As well as the afterimage test, they tested eye dominance with more conventional methods (Miles test and hole-in-the-card test). Only 16/30 dyslexics compared with 28/30 typical observers had a dominant eye on these measures. These results did not always agree with the afterimage test (which showed asymmetry in only 3/30 cases), and it was argued the conventional tests had ‘possible artefacts’. Nevertheless, the traditional eye dominance test results show a huge effect size of dyslexia, in contrast to prior studies [13].

The widespread evidence of phonological processing problems in dyslexia is hard to account for in terms of a peripheral visual mechanism. Le Floch & Ropars [1] state that asymmetry between the two eyes ‘should place a crucial role in the nervous connectivity for both the visual and the phonological processes in the different modal and cross-modal regions of the brain’, and cite literature to support the claim that dyslexia is ‘characterized by a weaker brain lateralization’. However, one of the references they cite concluded that differences in language lateralization between dyslexics and typical readers are small at best, and likely to be a consequence rather than a cause of impaired language learning [14]. There is also no indication that language lateralization relates to eye dominance.

The claim that ‘dyslexics confuse left and right and often make mirror errors in reading’ [1] is accompanied by citations to five empirical studies, but none of them supports this assertion. Most studied mirror errors in typical readers. For instance, Cornell [15] concluded regarding mirror-writing: ‘this phenomenon is a normal component of development and does not imply dysfunction’. The one study with a focus on dyslexia, by McCloskey & Rapp [16], described a single case of an adult with unusual visual localization problems, but these did not indicate specific problems with mirror errors—on the contrary, ‘d’ could be read as ‘b’, ‘p’ or ‘q’. This lack of accuracy in referring to prior literature does not give confidence in Le Floch & Ropars's scholarship or attention to detail.

Other claims in their discussion [1] are supported by information in the supplementary material that is so brief and incomplete as to be anecdotal. Thus, in support of a genetic origin of the dominance issue, we are presented with data from four family members of one dyslexic observer who also show no dominance on the two tests, but no information is given as to their reading abilities other than that a statement that they had dyslexia.

## 5. Overinterpretation of findings and potential conflict of interests

Le Floch & Ropars's conclusion [1] overinterprets the findings of the study, claiming a potential for ‘novel strategies to overcome reading and other neurological difficulties’ through the use of stroboscopic illumination that is purported to counteract confusing mirror images that affect reading, but the only evidence comes from a brief description in the supplementary material. Nothing in the data presented in the paper supports this idea and there is no logical connection between the proposed retinal anomalies and the suggestion that stroboscopic light could have any effect on dyslexia.

Despite those unsubstantiated conclusions and no independent replication of the findings obtained with the foveascope, the paper won a prize from the French academia of medicine in 2020 and received nine patent citations up to August 2023 [17] that gave rise to devices (lamps and glasses with a standardized scintillation) intended to treat dyslexia. In 2021, an eminent group of French dyslexia experts expressed concern about the lack of evidence for such stroboscopic interventions [18] that are being marketed in numerous countries. Le Floch & Ropars's paper [1] is cited as though it provides a rationale for the stroboscopic manipulation used in the devices. Although two clinical trials have been conducted, one for the lamps (NCT04157829) and one for the glasses (NCT04586621), their results have not been published in the peer-reviewed literature, nor are they posted on clinicaltrials.gov. We urge the private sponsors to communicate the results regarding their primary outcomes. Another clinical study (NCT05514457) is under way, with results expected in 2025. Meanwhile, independent evidence is starting to accumulate against the efficacy of stroboscopic manipulation. In a recent *Proceedings of the Royal Society B* paper, Lubineau *et al*. reported negative evidence regarding impacts on reading from four experiments testing impact of flickering illumination: the flickering light emitted by the glasses and lamp were not found to help dyslexic readers, beyond a moderate placebo effect [19]. These findings are further supported by a recent preprint of Lapeyre *et al*. that failed to identify any positive impact of pulsed lighting on the reading skills of adults with dyslexia [20].

## 6. Ethical issues

No institutional approval (IRB) is mentioned in the paper for the research study of Le Floch & Ropars [1]. This is at odds with the journal policy, which notes the need for committee approval for research involving human subjects. At 8 min and 19 s, the video attached in the supplementary material reads ‘The study were approved by Institutional Research Boards at the University of Rennes 1’. This is a misleading statement that deserves to be corrected, since, to our knowledge, there was no Institutional Research Board at the University of Rennes 1. In addition, we have no evidence that participants were given the kind of written information sheet routinely required for this kind of research, and suspect that the authors, as physicists, were unaware of usual ethical requirements for research with human subjects in the twenty-first century. Had they followed such procedures, they would be able to produce information sheets and consent forms, but these have not been made available. Last, we have concerns that affiliations of authors may confuse the readers. More specifically, the address given for the lab ‘Laboratoire d'Electronique Quantique et Chiralités' refers to a private house. It is unclear which scientific institution is responsible for this laboratory.

## 7. Conclusion

In sum, we have concerns about the publication of the paper by Le Floch & Ropars [1]. There is vagueness and inconsistency about key methodological details, such as whether testing was blinded, the characterization of the sample, and what instructions participants were given. Furthermore, the reported effect sizes, with visual dominance measures giving perfect discrimination between dyslexic and typical groups, are so extraordinary that they require extraordinary evidence to be believed, particularly since they are discrepant with an extensive prior literature. We feel this study is highly problematic and probably not reproducible, and that citing it to support advertising of commercial devices to remediate dyslexia is not justified.

## Data Availability

This article has no additional data.
